# Preclinical Evaluation of the Efficacy of Antivenoms for Snakebite Envenoming: State-of-the-Art and Challenges Ahead

**DOI:** 10.3390/toxins9050163

**Published:** 2017-05-13

**Authors:** José María Gutiérrez, Gabriela Solano, Davinia Pla, María Herrera, Álvaro Segura, Mariángela Vargas, Mauren Villalta, Andrés Sánchez, Libia Sanz, Bruno Lomonte, Guillermo León, Juan J. Calvete

**Affiliations:** 1 Instituto Clodomiro Picado, Facultad de Microbiología, Universidad de Costa Rica, San José 11501-2060, Costa Rica; gabriela.solano@ucr.ac.cr (G.S.); maria.herrera_v@ucr.ac.cr (M.H.); alvaro.seguraruiz@ucr.ac.cr (A.S.); mariangela.vargasarroyo@ucr.ac.cr (M. Vargas); mauren.villaltaarrieta@ucr.ac.cr (M. Villalta); andres.sanchez_b@ucr.ac.cr (A.S.); bruno.lomonte@ucr.ac.cr (B.L.); guillermo.leon@ucr.ac.cr (G.L.); 2 Instituto de Biomedicina de Valencia, CSIC, Valencia 46010, Spain; dpla@ibv.csic.es (D.P.); libia.sanz@ibv.csic.es (L.S.); jcalvete@ibv.csic.es (J.J.C.); 3 Sección de Química Analítica, Escuela de Química, Universidad de Costa Rica, San José 11501-2060, Costa Rica

**Keywords:** antivenoms, snake venoms, neutralization tests, preclinical efficacy, antivenomics, the 3Rs

## Abstract

Animal-derived antivenoms constitute the mainstay in the therapy of snakebite envenoming. The efficacy of antivenoms to neutralize toxicity of medically-relevant snake venoms has to be demonstrated through meticulous preclinical testing before their introduction into the clinical setting. The gold standard in the preclinical assessment and quality control of antivenoms is the neutralization of venom-induced lethality. In addition, depending on the pathophysiological profile of snake venoms, the neutralization of other toxic activities has to be evaluated, such as hemorrhagic, myotoxic, edema-forming, dermonecrotic, in vitro coagulant, and defibrinogenating effects. There is a need to develop laboratory assays to evaluate neutralization of other relevant venom activities. The concept of the 3Rs (Replacement, Reduction, and Refinement) in Toxinology is of utmost importance, and some advances have been performed in their implementation. A significant leap forward in the study of the immunological reactivity of antivenoms against venoms has been the development of “antivenomics”, which brings the analytical power of mass spectrometry to the evaluation of antivenoms. International partnerships are required to assess the preclinical efficacy of antivenoms against snake venoms in different regions of the world in order to have a detailed knowledge on the neutralizing profile of these immunotherapeutics.

## 1. Introduction

Snakebite envenoming is a neglected tropical disease that exerts a high burden of mortality and morbidity, particularly in impoverished rural regions of Africa, Asia, Latin America and parts of Oceania [1,2,3]. Administration of animal-derived antivenoms is the only scientifically-validated therapy for these envenomings ever since the development of the first serum antivenimeux by Albert Calmette, and following studies of [4,5]. The technology of antivenom manufacture experienced a series of improvements in the 20th century, with the introduction of fractionation protocols aimed at generating purified preparations of whole IgG or, alternatively, of the immunoglobulin fragments F(ab’)_2_ or Fab [6]. Currently, there is a heterogeneous universe of antivenom manufacturers in all continents, but limitations in antivenom availability and accessibility in various parts of the world, especially in sub-Saharan Africa and parts of Asia, constitute a significant public health problem [7,8,9].

In contrast to other immunoglobulin preparations used for passive immunization, such as tetanus antitoxin and anti-rabies immunoglobulins, in which the antigens to be neutralized by antibodies present little variability regardless of geographical location, antivenoms for snakebite envenomings differ in their specificity depending on the particular venoms being used for immunization. There is a large variation in snake venom composition at all taxonomic levels, including genera, species, and even between and within populations and individuals of the same species [10,11]. Moreover, a complex pattern of ontogenetic shifts in venom composition has been described for the venoms of some species, hence opening another level of variation [12,13,14,15]. Thus, despite the fact that snake venoms are generally constituted by a limited number of protein families, there are variations in the proportion of each type of protein family in different venoms and, within a single protein family, there are many iso- and proteoforms with distinct toxicological and immunological profiles [16,17,18]. Elapid snake venoms are predominantly composed of neurotoxins or cytotoxins of the three-finger toxin family and phospholipases A_2_ (PLA_2_) [19,20,21]. Viperid venoms, in contrast, are composed mostly of PLA_2_s, zinc-dependent metalloproteinases (SVMPs) and serine proteinases (SVSPs) [22]. Venoms of stiletto snake species of the subfamily Atractaspidinae, are rich in sarafotoxins [23], whereas those of species of the highly diverse non-front-fanged group of the family Colubridae contain variable sets of protein families [24]. In addition to these predominant protein types, venoms of the various snake families contain other components that, albeit generally minor, may play key roles in toxicity, such as C-type lectin-like proteins, disintegrins, vasoactive peptides, hyaluronidase, cysteine-rich secretory proteins (CRISPs), and low molecular mass myotoxins among others [22,25]. 

Such biochemical, and consequently immunological, diversity constitutes a great challenge for antivenom design, manufacture and quality control. An antivenom effective against a group of venoms in a particular country may not be effective against other venoms of relevance in the same country, or against venoms of species from another region. This phenomenon has had a significant impact in public health since, owing to deficient regulatory control, some antivenoms have been exported to countries where they are not effective (see for example [26]). Frequently, antivenoms have been introduced to countries without a robust preclinical evaluation of their efficacy against the medically most relevant venoms [8,27]. The design of venom mixtures for immunization aimed at antivenom production has been largely based on empirical criteria and not on a rigorous evaluation of the best venom combinations. The flourishing field of proteomics has allowed a comprehensive and in depth knowledge of the composition of many snake venoms (venomics), including some of the medically most important species. In parallel, an increased understanding on the immunological relationships of the various venom components has been also reached, by the combination of neutralization studies and “antivenomics”, a field of research that harnesses the potential of proteomics for assessing the immunoreactivity of antivenoms [25,28]. 

The complexity of snake venoms, and the consequent wide spectrum of pathological and pathophysiological manifestations of envenomings due to the concerted actions of several toxin types, represents a great challenge for the preclinical evaluation of the efficacy of antivenoms, as a prior condition to the clinical evaluation of their safety and efficacy. The present review summarizes the state-of-the-art in the field of preclinical evaluation of antivenoms, and presents some of the unsolved issues that require further studies and developments. 

## 2. Neutralization of Lethality: The Gold Standard in the Preclinical Assessment of Antivenom Efficacy

Being lethality the ultimate consequence of snakebite envenoming, the neutralization of this effect became the centerpiece of the evaluation of the preclinical efficacy of antivenoms since the dawn of this immunotherapy (see [29,30]). Generally, mice are used in these tests [31,32], although some laboratories work with other species, such as guinea pigs [32]. Initially, the Median Lethal Dose (LD_50_) of a particular venom is determined by administering various doses to groups of mice and recording the number of dead animals within a predetermined time interval, usually 24 h or 48 h. LD_50_ is then estimated by Probits [33], Spearman–Karber [34], or non-parametric tests [32]. For the assessment of neutralization of lethality, a “challenge dose” of venom is selected, corresponding to a number of LD_50_s (which usually range between 3 and 6 depending on the laboratory). Then, mixtures containing a fixed concentration of venom and variable dilutions of the antivenom are prepared, and incubated. Afterwards, aliquots of the mixtures, containing the challenge dose of venom, are injected in mice using the same route of administration employed for the determination of the LD_50_. A control group of animals receives the same dose of venom incubated with saline solution or with a solution containing albumin. Deaths occurring within a specified time interval are recorded and neutralization is expressed as Median Effective Dose (ED_50_), i.e., the volume of antivenom or the venom/antivenom ratio at which 50% of injected mice survived. Some quality control laboratories express the neutralizing efficacy in terms of “potency” [35]. 

There are many variations in the way this test is performed in different laboratories, and this introduces difficulties when comparing data coming from various sources. These variations include: (a) The number of LD_50_s used as challenge dose. This has implications since the higher the number of LD_50_s in the challenge dose, the lower the estimated antivenom efficacy [36]. Moreover, in some cases, the challenge dose is defined as the Minimum Lethal Dose (MLD) [37]. (b) The strain and weight of mice used in the tests, as some studies have revealed variations in the susceptibility of certain strains to venoms [38,39]. (c) The route of venom injection. Most laboratories use the intravenous (i.v.) route in the estimation of LD_50_ and ED_50_ [32], although others, especially in Latin America, use the intraperitoneal (i.p.) route [35,40]. Different pathophysiological mechanisms, and hence different toxins, may predominate in the overall toxicity depending on the route of injection (see below). (d) The ways in which neutralization is expressed, that could be µL antivenom per challenge dose of venom, mg venom per mL antivenom, µL antivenom per mg venom, or mg antivenom per mg venom, and even the number of LD_50_s neutralized per mL antivenom, although the latter has been criticized and is not recommended in the revised version of the WHO Guidelines for Production, Control and Regulation of Snake Antivenom Immunoglobulins [41]. It is necessary to characterize in greater detail the design, analytical properties, and factors affecting the outcome of these tests, since few studies have been performed on this important topic [31,39,42,43]. 

Some works have approached the neutralization of lethality by using a protocol that resembles the natural setting of snakebite more closely, i.e., by administering the antivenom after envenoming. This type of “rescue protocol” involves the injection of a challenge dose of venom, followed by the i.v. injection of various doses of antivenom [31,44,45]. ED_50_ is defined as the volume of antivenom which protects 50% of injected mice. Despite its valid rationale, this protocol has not been introduced into quality control laboratories because these tests are more difficult to standardize, and also since, in this context, results are influenced by venom toxicokinetics and antivenom pharmacokinetics, and not just the intrinsic antivenom neutralizing capacity [46]. However, despite not being implemented in the routine quality control procedures, the information provided by this type of assessment is valuable to understand neutralization in the context of the dynamics of envenoming (see Section 7). 

## 3. The Need to Understand the Mechanisms of Toxicity in the Lethality Assay

Being the determination of LD_50_ and ED_50_ of such paramount relevance in the analysis of antivenom preclinical efficacy, it is noteworthy that relatively few studies have addressed the predominant mechanisms of toxicity in these tests and, hence, the venom activities that are being assessed, vis-à-vis the pathophysiology of envenomings in the clinical setting. In the case of neurotoxic venoms, such as those of elapid and some viperid species, clearly the cause of death is neuromuscular paralysis and respiratory failure secondary to the action of pre- and/or post-synaptically acting neurotoxins [47]. However, in other venoms, the situation is more complex. Procoagulant venoms, such as those of many viperids and some elapids, are likely to cause death by fulminant intravascular thrombosis when injected by the i.v. route [48,49,50]. In contrast, when using the i.p. route, viperid hemorrhagic venoms cause a massive extravasation leading to cardiovascular collapse, as shown for the venom of *Bothrops asper* [51]. It is necessary to gain a better understanding on the main toxicity mechanisms of medically-relevant venoms in the experimental conditions in which lethality and neutralization tests are performed.

## 4. The Complex Pathophysiology of Snakebite Envenoming Demands a Wider Approach for the Preclinical Testing of Antivenoms

Snakebite envenomings generally involve a rather complex pathophysiology, owing to the great variety of toxins present in most venoms, as revealed by proteomic analysis [25]. Many species of the family Elapidae, such as the mambas (*Dendroaspis* sp.), kraits (*Bungarus* sp.), coral snakes (*Micrurus* sp.), and many cobras (*Naja* sp.) predominantly induce a neurotoxic effect secondary to the blockade of neuromuscular transmission due to the action of neurotoxins [47]. In these cases, the study of the neutralization of lethality is a good model to assess the preclinical efficacy of antivenoms. However, in many other species, different pathological and pathophysiological activities of the venoms mediate in the overall toxicity in humans and should be considered in the evaluation of antivenoms.

Envenomings by sea snakes, some terrestrial elapid species and the South American rattlesnake are characterized by systemic myotoxicity, i.e., rhabdomyolysis, in addition to neurotoxicity [52,53]. Such massive muscular damage often causes acute kidney injury secondary to the deposition of large amounts of myoglobin in the renal tubules. Many cobra venoms, such as those of the spitting species in Africa and Asia, cause a predominantly local necrotizing effect in humans [54]. A number of elapid species of Australia and Papua New Guinea contain, in addition to potent neurotoxins, prothrombin activators that cause significant hemostatic disturbances and, in some cases, myotoxic PLA_2_s that cause systemic myotoxicity and acute kidney injury [55]. The majority of viperid venoms, on the other hand, induce local tissue damage (myonecrosis, dermonecrosis, hemorrhage, and edema) and systemic alterations associated with coagulopathies, bleeding in various organs, hemodynamic disturbances, and renal alterations [47,54,56]. Some of these effects may not result in lethality, but cause tissue damage that may end up in permanent sequelae. In all these cases it is clear that the assessment of the neutralization of lethality by antivenoms is insufficient for an integrated evaluation of antivenom preclinical efficacy, and a wider set of neutralization assays is required. This view was incorporated in the WHO Guidelines for Antivenoms, in which “essential” and “recommended” tests are described for the preclinical assessment of antivenoms [32]. The evaluation of neutralization of lethality was defined as the “essential” preclinical test, but supplementary tests, aimed at evaluating the neutralization of other toxic activities, are recommended for new antivenoms and for introducing existing antivenoms into new geographical settings [32]. These supplementary tests include assays for evaluating the neutralization of hemorrhagic, necrotizing, in vitro procoagulant, in vivo defibrinogenating, myotoxic, and neurotoxic activities, although in the WHO Guidelines it is mentioned that the lethality test is reliable to predict neutralization of neurotoxicity [41]. A brief description of the methodologies used for assessing these effects follows.

### 4.1. Hemorrhagic Activity

The assessment of the neutralization of hemorrhagic activity is important for most viperid venoms, and also for some non-front fanged colubrid venoms of potential relevance in human envenomings, such as those of *Dispholidus typus* and *Rhabdophis* sp. Since its development in 1960, the rodent skin assay to assess hemorrhagic activity has become widely used for quantifying this effect and its neutralization. The test was originally developed in rabbits [57], although most groups currently use either rats [58] or, more often, mice [59]. Hemorrhagic activity is determined by injecting variable amounts of venom intradermally in the abdominal region of the animals. After a time interval, usually 2 h, animals are sacrificed, their skin removed, and the size of the hemorrhagic lesion in the inner side of the skin is measured. Hemorrhagic activity is expressed as the Minimum Hemorrhagic Dose (MHD), defined as the dose of venom that induces a hemorrhagic area of 10 mm diameter [59]. One limitation of this procedure is that similar hemorrhagic areas might show different intensities of hemorrhage. This can be circumvented by measuring the amount of hemoglobin present in the hemorrhagic lesion [60]. For the study of neutralization by antivenoms, a challenge dose of venom corresponding to five MHDs is used, and neutralization is expressed as ED_50_, i.e., the volume of antivenom, or the venom/antivenom ratio, in which the size of the hemorrhagic spot is reduced by 50% [59]. 

### 4.2. Necrotizing Activity

Neutralization of necrotizing activity in the skin, i.e., dermonecrosis, is relevant in the case of venoms that induce cutaneous necrosis, such as those of spitting cobras (*Naja* sp.) of Africa and Asia [54,61]. The method is performed in rodents, usually rats or mice, and is based on the intradermal injection, in the abdominal region, of solutions containing various venom doses. After 72 h, animals are sacrificed, their skin removed, and the size of the necrotic lesion in the inner size of the skin measured [58,62]. The Minimum Necrotizing Dose (MND) corresponds to the dose of venom inducing a necrotic lesion of 5 mm diameter. For neutralization studies, a challenge dose of one MND is used [62]. Neutralization is expressed as ED_50_, i.e., the volume of antivenom, or the venom/antivenom ratio, in which the size of the necrotic lesion is reduced by 50%.

### 4.3. In Vitro Coagulant Activity

Many snake venoms are procoagulant when added to plasma, by acting on several factors of the coagulation cascade. In clinical cases, the most important consequence of this effect is consumption coagulopathy and defibrinogenation, which may contribute to profuse bleeding [63]. The assessment of the neutralization of this effect by antivenoms is important for many viperid and some Australian elapid venoms. In vitro procoagulant activity is usually tested in human citrated plasma or in solutions of bovine fibrinogen [58]. When using plasma, the test detects several types of procoagulants, such as thrombin-like enzymes, and activators of factors X and prothrombin. When using fibrinogen solutions, the assay detects only thrombin-like enzymes. Thus, for antivenom efficacy assessment the use of citrated plasma is generally preferred. Various doses of venom are added to plasma, or fibrinogen, previously incubated at 37 °C, the mixtures are kept at 37 °C, and the time when a firm clot is formed is recorded. Activity is expressed as the Minimum Coagulant Dose-plasma (MCD-P), or the Minimum Coagulant Dose-fibrinogen (MCD-F), i.e., the amount of venom that induces clotting of plasma, or fibrinogen, in 60 sec [58,64]. For neutralization studies, a challenge dose of two MCDs is used [64]. Neutralization is expressed as Effective Dose (ED). Some authors define ED as the volume of antivenom, or the venom/antivenom ratio, at which clotting is increased three times as compared to clotting times of plasma, or fibrinogen, incubated with venom alone [64]. Another way to express ED is the lowest volume of antivenom which completely prevents clotting [32].

### 4.4. Defibrinogenating Activity

In general, snake venoms that induce in vitro clotting of plasma are able to cause defibrinogenation in vivo. This activity is assessed by injecting various doses of venom i.v. in mice. One hour after envenoming, animals are bled by cardiac puncture or from the orbital venous plexus, under anesthesia. Blood is placed in glass tubes and left undisturbed for 1 h at room temperature. At the end of this period, tubes are gently tilted and the formation of a clot is observed. Activity is expressed as the Minimum Defibrinogenating Dose (MDD), defined as the lowest venom dose which renders blood unclottable in all mice injected; blood from all control mice receiving saline solution instead of venom should clot [58,64]. For the study of neutralization, a challenge dose of two MDDs is used [64]. Neutralization is expressed as Effective Dose (ED), i.e., the minimum volume of antivenom, or the venom/antivenom ratio, at which all blood samples clot after 1 h incubation [41,64].

### 4.5. Myotoxic Activity

Many venoms of species of the families Elapidae and Viperidae induce local and, in some cases, systemic myotoxicity, which is mostly caused by PLA_2_s [65]. Some elapid venoms, such as those of sea snakes and Australian terrestrial species, and the venoms of few viperids, such as the South American rattlesnake, induce systemic myotoxicity which may in turn contribute to acute kidney injury [55,66]. Many viperid venoms cause local myonecrosis [65]. Venom-induced myotoxicity can be studied by histological analysis of affected muscle tissue. Quantitatively, it is feasible to estimate the extent of myonecrosis by quantifying, in tissue sections, the total number of fibers and the number of necrotic fibers, which are usually identified by the presence of hypercontraction of myofibrils. Thus, a Necrotic Index can be obtained by dividing the number of necrotic fibers by the total number of muscle fibers [67]. However, since histological analysis is time consuming and not all quality control laboratories have facilities to process tissues, an alternative assay is the quantification of the plasma or serum activity of the enzyme creatine kinase (CK), which is released from damaged muscle fibers to the circulation [68]. Various venom doses are injected intramuscularly (i.m.) in the gastrocnemius or thigh muscles of mice and, after 3 h, a blood sample is collected and the CK activity of plasma or serum is determined. The Minimum Myotoxic Dose (MMD) is the amount of venom that increases four times the CK activity, as compared to mice injected with saline solution [32,69]. For neutralization, a challenge dose of 3 MMDs is used, and neutralization is expressed as Median Effective Dose (ED_50_), i.e., the volume of antivenom, or the venom/antivenom ratio, at which CK activity is reduced by 50% as compared to that of mice injected with venom alone [69].

### 4.6. Edematogenic Activity

Edema at the site of venom injection is characteristic of envenomings by vipers, and by some cytotoxic elapids [54,61]. This effect can be studied experimentally by injecting various doses of venom subcutaneously in the footpad of mice or rats. Then, at various time intervals, the increment in the volume of the pad is assessed by either plethysmometry [70,71] or by measuring the thickness of the injected pad with a low-pressure spring caliper [72]. These two techniques have largely replaced older methods based on determining the weight of footpads of mice after euthanasia [73] since they only allow the determination of edema at one time interval. The Minimum Edematogenic Dose (MED) is the amount of venom that induces an increment of 30% in footpad volume or thickness 1 h after injection [69]. For neutralization, a challenge dose of six MEDs has been used, and antivenom efficacy (ED_50_) is the volume of antivenom, or venom/antivenom ratio, which reduces by 50% the edema induced by venom alone [69]. 

### 4.7. Neurotoxic Activity

**Figure 1:**
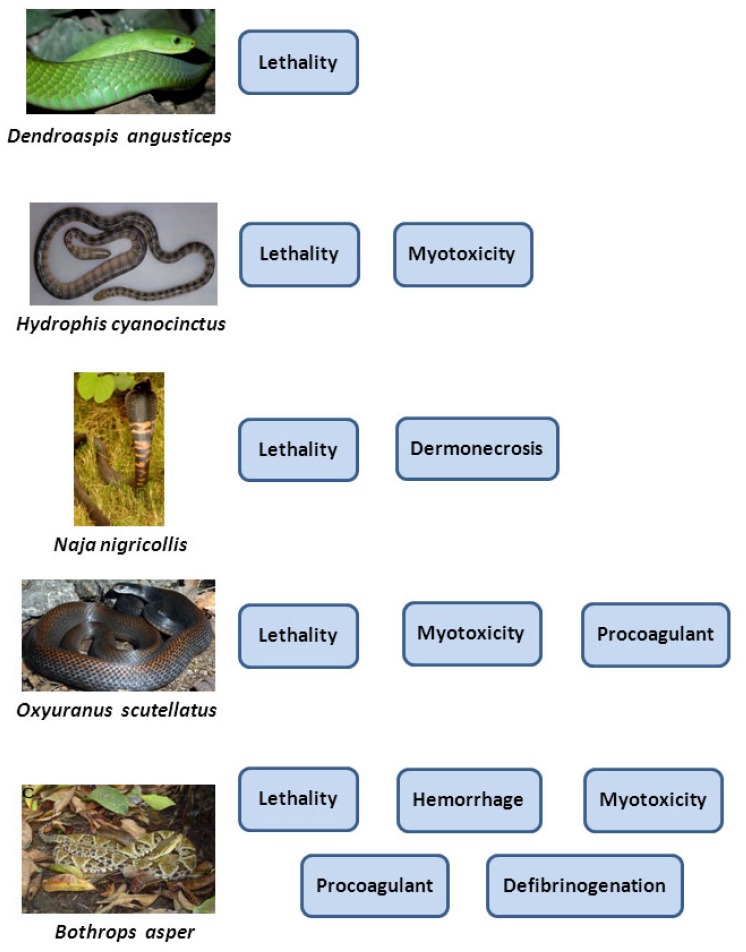
Most relevant toxic activities induced by venoms of different snake groups that need to be considered in the preclinical evaluation of antivenoms. Venoms of many elapid species, and few viperids, mainly induce neurotoxicity, which is assessed by lethality. Sea snake venoms induce both neurotoxicity and systemic myotoxicity. The main effect in envenomings by spitting cobras is cutaneous necrosis, which has to be evaluated in addition to lethality. Several Australian terrestrial elapids, as well as few rattlesnake venoms, induce neurotoxicity, myotoxicity and consumption coagulopathy, which depends on their coagulant enzymes. Envenomings by many viperid species cause, in addition to lethality, local tissue damage (hemorrhage and myotoxicity), and systemic effects associated with bleeding and coagulopathy. Other pathophysiological alterations that would need to be considered in the preclinical evaluation of antivenoms are thrombocytopenia, platelet hypoaggregation, acute kidney injury, and systemic vascular capillary leakage syndrome. Reproduced from [46], copyright 2013 Elsevier.

Various methods have been implemented to evaluate the inhibitory effect of snake venoms on neuromuscular preparations ex vivo [74,75,76,77]. The time to reach either 50% or 90% neuromuscular blockade is estimated. Neutralization studies are performed either by incubating venom and antivenom before addition to the bathing solution or by first adding the venom to the medium, and then applying various volumes of antivenom [78,79]. ED_50_ corresponds to the volume of antivenom, or the venom/antivenom ratio, at which the neuromuscular blockade is reduced by 50%. This methodology requires equipment and expertise which make it difficult to perform in routine quality control laboratories. Hence, it is considered that the neutralization of lethality, in predominantly neurotoxic venoms, is directly related to the neutralization of neurotoxicity [32]. Figure 1 summarizes the most important effects that need to be tested for various types of snake venoms for the preclinical evaluation of antivenom efficacy. 

## 5. Towards Expanding the Set of Laboratory Tests for the Evaluation of Antivenoms

In addition to the assays described above, there are other toxicological effects which play a relevant role in snakebite envenomings and whose neutralization by antivenoms is important to assess. Examples are: (a) Cardiovascular alterations: Some studies have monitored the effects of venoms on the mean arterial blood pressure after i.v. injection of venoms [80,81]. Alternatively, the increments in the blood lactic acid concentration might be used to monitor the increment in anaerobic metabolism as a consequence of reduced blood flow and ischemia in tissues [80]. (b) Thrombocytopenia and platelet hypoaggregation: Alterations in platelet counts and function are important in envenomings by some viperid species [82,83]. Thrombocytopenia can be assessed by administering various venom doses i.v. and then, at 1 h, determining platelet counts in blood. The Thrombocytopenic Dose 50% corresponds to the amount of venom that reduces blood numbers by 50% [84]. Likewise, platelet hypoaggregation can be assessed by preparing platelet-rich plasma from envenomed animals, and assessing aggregation after addition of agonists such as ADP or collagen [84,85]. (c) Acute kidney injury induced by a number snake venoms: Studies have been performed using histological assessment of renal damage, alterations in functional parameters renal perfusion system [86], and quantification of concentration of metabolites in serum, such as creatinine, which are elevated in kidney injury [87]. Other clinically-relevant effects for which laboratory tests have to be implemented are the thrombotic activity characteristic of envenomings by the Caribbean species *Bothrops lanceolatus* and *B. caribbaeus* [88], the systemic leakage syndrome described in envenomings by some populations of *Daboia russelli* [61], the hyponatremia described in envenomings by *Bungarus multicinctus* [89], and the cardiotoxic effect induced by the venom of stiletto snakes of genus *Atractaspis* [90]. It is necessary to standardize laboratory procedures for modeling these and other venom activities and for assessing the neutralizing ability of antivenoms. 

## 6. The Issue of Minimum Accepted Values of ED_50_ for Preclinical Neutralization Tests

There is a lack of international consensus on the acceptable limits for the preclinical neutralizing ability of antivenoms other than the general concept that they have to neutralize the most relevant toxic activities of the venoms they are supposed to neutralize. Thus, when evaluating an antivenom preclinically, the first step seems to be a yes/no decision on whether an antivenom neutralizes a particular effect, i.e., lethality, hemorrhagic activity, coagulant activity, etc. An antivenom which proves ineffective for neutralizing a clinically-relevant toxic effect of a venom should not be accepted for its clinical use. However, even in the case where neutralization is achieved, there are huge differences in the values of ED_50_s between antivenoms. For example, when assessing the efficacy of antivenoms distributed in sub-Saharan Africa in the neutralization of toxic effects of the venom of *Echis ocellatus*, the majority of the antivenoms tested were able to neutralize the effects, but with wide variations in their ED_50_. The case of neutralization of in vitro coagulant activity illustrates this point. When expressed in terms of mg venom neutralized per mL antivenom, the most effective antivenom had an ED_50_ of 12.88 ± 0.09 mg/mL, whereas the antivenom with the lowest efficacy had an ED_50_ of 0.1 ± 0.01 mg/mL, a 128 fold difference [91]. 

The majority of antivenoms raised against elapid venoms, which predominantly contain antigenically-weak low molecular mass neurotoxins and PLA_2_s, have values of ED_50_ for the neutralization of lethality ranging from 0.5 mg/mL to 1.5 mg/mL, although there are cases in which higher potencies are achieved [50,92]. In contrast, antivenoms against viperid venoms, whose most relevant toxins are SVMPs, serine proteinases and PLA_2_s, often have potencies of 2 mg/mL or above [40], although there are antivenoms with lower potencies being distributed [93]. Some Pharmacopoeias establish the potency values that antivenoms should have, but nevertheless there is a lack of regulatory framework for antivenoms in many countries.

Several antivenoms used for the treatment of envenomings by *Bothrops* sp. in Latin America have ED_50_s of 3 mg/mL or higher for lethality [40]. This has been established in a number of countries in the region as the minimum acceptable level for antivenoms. In Australia, there is a required potency for the various antivenoms used (defined as units per vial) [53]. However, there are other regions in the world, such as sub-Saharan Africa, in which no parameters for antivenom acceptance have been defined yet. Consequently, antivenoms of highly variable potencies are distributed [91,93]. Hence, there is a need to establish acceptable limits of potency of antivenoms at the preclinical level, a task that demands the concerted work of laboratory scientists, clinical researchers, regulatory agencies, antivenom manufacturers, and the WHO and its regional offices. Regional workshops involving these stakeholders are required to define the acceptable potencies for antivenoms and the preclinical tests that should be performed before an antivenom is introduced for clinical use.

## 7. The Impact of Venom Toxicokinetics and Antivenom Pharmacokinetics in the Effectiveness of Antivenoms: Pharmacokinetic-Pharmacodynamic Relationships

As discussed above, the most common way to test antivenom efficacy at the preclinical level is based on a protocol that involves incubating venom and antivenom before testing in the in vitro or in vivo assays described. However, this experimental design does not take into account the complex pharmacokinetic-pharmacodynamic interactions involved in venom neutralization in vivo. Several groups have studied the neutralization of snake venoms using a protocol that involves the independent injection of venom and antivenom, thus simulating the real circumstances of snake bites in which pharmacokinetic parameters are critical. The results obtained in these studies highlight relevant aspects of venom neutralization since there is frequently a mismatch between the toxicokinetics of venom components and the pharmacokinetics of antivenom antibodies or antibody fragments. Toxins and antibodies usually have different volumes of distribution and vary in the time needed to reach equilibrium in their distribution space [94]. Likewise, venom toxins act on their targets in the tissues or blood at variable time-courses. 

One aspect of snakebite envenoming in which this mismatch is notorious has to do with the action and neutralization of toxins inducing local tissue damage in viperid venoms and cytotoxic elapid venoms [95]. Once injected in a natural bite, the toxins responsible for local pathology, especially hemorrhagic SVMPs, myotoxic PLA_2_s, and cytotoxic three-finger toxins act very rapidly and induce skeletal muscle necrosis, degradation of extracellular matrix, dermonecrosis, and hemorrhage within minutes of injection [96,97]. Therefore, even when antivenoms are administered by the i.v. route immediately after envenoming, the neutralization of these local effects is only partial [59,98,99]. In these circumstances, the distribution of antivenom antibodies to the affected tissue is limited, and the deleterious action of the toxins occurs rapidly. This is the case even when using antivenoms able to neutralize these local effects when incubated with venom prior to injection [95,98]. Furthermore, local i.m. injection of antivenom at the site of tissue damage did not improve the neutralization as compared to i.v. administration [98], probably due to constraints in the diffusion of antibodies in a damaged tissue environment. 

When similar, “rescue-type” experiments have been performed to assess the neutralization of neurotoxicity, i.e., lethality, induced by elapid venoms, a rapid i.v. administration of antivenoms after envenoming prevents death. However, effectiveness of antivenoms decreases as the time lapse between envenoming and antivenom administration increases [45,79,100]. There is, therefore, a window of time when antivenom antibodies are able to bind toxins in the circulation before they reach their targets at the neuromuscular junctions. Thus, even when this type of “rescue” experiments are not routinely performed in the preclinical assessment of antivenoms, they bring valuable information regarding the dynamics of envenoming and therapy, and raise research issues related to the need of alternative ways to improve snakebite envenoming treatment such as, for example, the search for natural and synthetic inhibitors that could circumvent some of the limitations of antibodies, particularly regarding local tissue damage [101].

## 8. Implementation of the 3Rs in Antivenom Testing

The need to use animals in the evaluation of antivenoms, and the inherent suffering that venoms induce in these tests, have raised concerns about the need to reduce both the number of animals used and the pain associated with venom toxicity and neutralization tests. The WHO guidelines of antivenoms [32] place special emphasis on this aspect, and efforts by several groups are being performed to promote the concept of the 3Rs (Replacement, Reduction, and Refinement) in antivenom development and quality control.

The search for in vitro tests that would substitute in vivo assays, such as lethality and determination of hemorrhagic, myotoxic, dermonecrotic, and defibrinogenating activities, has been difficult due to the complex biochemical and toxicological nature of snake venoms, whereby toxicity is often determined by several toxins acting together in synergistic and additive fashions [102,103]. For neurotoxic venoms, the use of ex vivo neuromuscular preparations is useful for assessing neuromuscular blockade and its neutralization [79,104,105]. However, this procedure is difficult to implement and is usually performed in specialized laboratories and not in antivenom development and quality control laboratories. In some cases, in vitro immunochemical and enzymatic tests have shown good correlation with the neutralization of lethality. Examples are enzyme immunoassays for some viperid venoms [106,107]. In this case, the development of assays to assess the antibody titers against the proteins responsible for the main toxicity in a venom is likely to give better results than using the crude venom. Two cases that illustrate this principle are the venom of the South American rattlesnake *Crotalus durissus terrificus*, where toxicity largely depends on the neurotoxic and myotoxic action of the heterodimeric PLA_2_ complex crotoxin [105], and the venom of some neurotoxic elapids, such as *Naja kaouthia*, where toxicity depends mostly on the action of few α-neurotoxins [108]. For predominantly neurotoxic venoms, the Toxicity Score of venom components is a useful tool [109] which includes proteomic and functional analyses of fractions, a field known as Toxicovenomics [110]. Other in vitro immunological assays used to assess antivenom reactivity against venoms include size-exclusion chromatography [111] and turbidimetry [112]. The advent of “antivenomics”, with its powerful analytical potential, has constituted a significant leap forward in the in vitro preclinical assessment of antivenoms, and is discussed in the next section of this review.

For *Bothrops asper* venom, the neutralization of in vitro activities, such as PLA_2_ and procoagulant effects, has been shown to correlate with neutralization of lethality [51,113]. Thus, despite the fact that it is difficult to substitute the lethality test in the final quality control of antivenom efficacy, these surrogate tests may greatly reduce the number of mice used for following the development of antibody titers in horses, or help in reaching a preliminary yes/no decision on whether an antivenom is effective against a venom. Likewise, the second edition of the WHO guidelines on antivenoms presents the use of immunochemical tests, such as ELISAs or antivenomics, as screening tests of antivenom efficacy before using animals. Each laboratory should develop its own standards and limits of acceptance, mainly based on studies of correlation between these in vitro assays and the in vivo toxicity tests [41].

Effects other than lethality can be also substituted by in vitro assays. The myotoxic activity of snake venom PLA_2_s and PLA_2_ homologs, as well as of cytotoxins of the three finger toxin family, depends on their ability to directly attack the integrity of the plasma membrane of skeletal muscle fibers [65,114]. Thus, the use of myoblast/myotube cell culture systems is a suitable way to assess the myotoxic activity of venoms and myotoxins and its neutralization by antivenoms [115,116]. Likewise, renal toxicity by venoms is partially dependent on their cytotoxic effects on renal cells [86], although the pathophysiology of acute kidney injury in these envenomings also involves hemodynamic alterations and intravascular coagulation [117]. On the other hand, the hemostatic alterations of many venoms, associated with a consumption coagulopathy and defibrinogenation, is largely due to the action of procoagulant venom components on fibrinogen or coagulation factors II, V or X [63]. Thus, in vitro estimation of the clotting activity of venom on human citrated plasma [58,64] is likely to be a good surrogate test for defibrinogenating activity. In contrast, it has been difficult to develop in vitro assays that correlate with hemorrhagic activity since, although hemorrhage depends on proteinase action of SVMPs, there is no correlation with hemorrhage when this activity is tested on commonly used substrates, such as azocasein. It is suggested that physiologically-relevant substrates, such as type IV collagen, should be tested, owing to the relevance of hydrolysis of this basement membrane component in the pathogenesis of microvessel damage [118]. The use of chicken embryos at a developmental stage prior to the development of pain sensitivity has been proposed for studying neutralization of hemorrhagic activity of venoms [119].

“Humane” tests for assessing lethality and its neutralization have been developed. Preliminary dose-finding tests, using one mouse per dose, are recommended when first assessing the toxicity of a venom [32]. This allows the selection of appropriate range of venom doses for estimating the LD_50_. Likewise, some methods establish a final observation time of 8 h, after which all mice are euthanized. Depending on the condition of the animals, mice showing signs of severe envenoming are euthanized at any time before 8 h [120]; this reduces the time of observation and, therefore, the suffering. Caution should be exercised, nevertheless, when using this method, as observations in our laboratory with several venoms indicate that different results are obtained with this method as compared to a final observation time of 24 h (our unpublished data).

**Figure 2:**
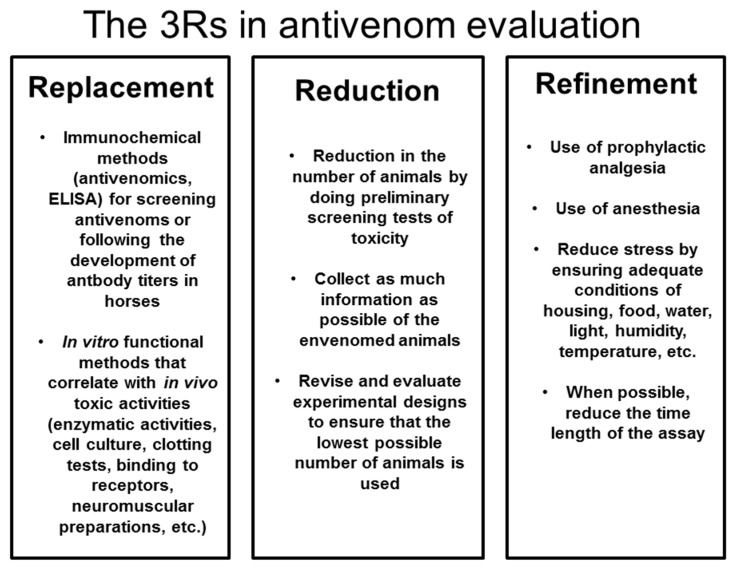
Possibilities for implementing the 3Rs in antivenom preclinical assessment. Some examples of interventions that have been developed or are being implemented in Toxinology laboratories to replace, reduce and refine the assays using animals are described.

Another important consideration is the use of analgesia, especially in venoms that induce severe pain associated with tissue damage, such as most viperid venoms and some elapid venoms [121]. A study with *Bothrops asper* venom demonstrated that the prophylactic use of the analgesics morphine or tramadol do not affect the outcome of the determination of lethal, hemorrhagic, myotoxic, edema-forming and defibrinogenating activities [51,122]. It is necessary to validate the use of these and other analgesics against different venoms, both in terms of their analgesic potential and assessing whether they do not affect the results of toxicity tests. Figure 2 depicts some of the possibilities of implementing the 3Rs concept in the preclinical evaluation of antivenoms.

## 9. Antivenomics: Harnessing the Analytical Potential of Proteomics for the Evaluation of Antivenoms

The understanding of the composition of snake venoms has grown exponentially in the last decade owing to the use of mass spectrometry-based methodologies to investigate venom proteomics [123,124]. A large number of venoms have been analyzed using this analytical platform and a fascinating view of the complexity and inter- and intra-species variability has emerged [20,125]. This explosive volume of information has enriched the study of venoms from both biological and medical perspectives, and has identified hitherto unknown venom components whose structures and actions need to be investigated [22,25]. 

A straightforward application of the body of knowledge gained through the venomics platform is the analysis of the immune reactivity of antivenoms against venoms, a field coined “antivenomics” [126]. Antivenomics is translational venomics, a proteomics-based protocol to quantify the extent of cross-reactivity of antivenoms against homologous and heterologous venoms. In its current format, the so-called “second generation antivenomics” platform [127] (Figure 2), antivenom molecules, whether whole antibodies or antibody fragments, are covalently immobilized onto a chromatographic matrix, the venom to be analyzed is pumped through the immunoaffinity column, and the unbound material washed clear prior to elution of the retained venom molecules by alteration of the mobile phase conditions that weaken the antibody-antigen interaction. Non-retained and retained protein mixtures are then separated by reverse phase HPLC using the same conditions employed for the proteomic analysis of the crude venom. Quantitative comparison of reverse-phase HPLC chromatograms of whole venom and the immunoaffinity column eluates provides thus qualitative and quantitative information on both the set of toxins bearing antivenom-recognized epitopes and those toxins exhibiting poor immunoreactivity. For each chromatographic fraction, the percentage of retained protein can be estimated by the equation:

% retained proteins = [retained/(retained + non-retained)] × 100

Maximum binding capacity of an antivenom for the different venom antigens can be estimated through a series of experiments where the amount of venom pumped through the immunoaffinity column is varied to estimate the µg of each venom component per mg antivenom protein that saturates their specific antibody binding sites [91]. 

Despite its recent introduction, the usefulness and validity of antivenomics to complement the in vivo standard preclinical assays of neutralization of lethality and toxic activities by homologous and heterologous antivenoms has been extensively demonstrated in a growing number of applications aimed at assessing the immunological profiles of antivenoms from different manufacturers and countries (see for example [91,93,128,129,130,131,132]). The combination of antivenomics and in vivo and in vitro neutralization tests constitutes thus a powerful toolbox that provides a robust evaluation of antivenom preclinical efficacy [128]. In this regard, the WHO guidelines of antivenoms highlights that antivenomics may be used as a way to assess whether an antivenom has the potential to be effective against a particular venom, before initiating in vivo toxicity tests, as a way to reduce the use of mice in the preclinical testing of antivenoms [41].

**Figure 3:**
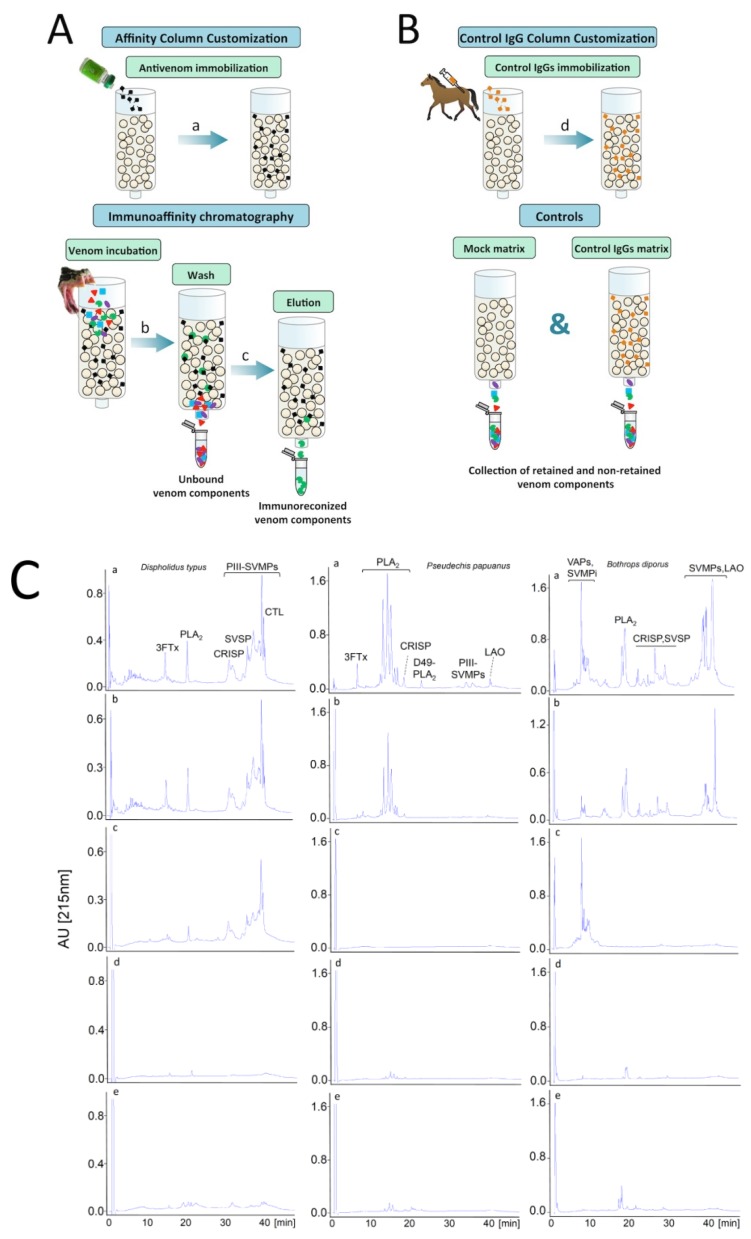
Cartoon of the “second generation” antivenomics workflow [127]. Panels (**A**) and (**B**) illustrate, respectively, the generation of the immunoaffinity (a) and control antibody (d) columns, and the steps of the antivenomic protocol to assess which toxins show immunoreactivity towards the immobilized antivenom molecules (c) and which do not bind to the immunoaffinity column (b). Mock matrix and control IgG columns, run in parallel to the immunocapture experiment, serve as specificity controls. Panel (**C**) displays three immunoaffinity experiments using venoms of snakes from different families: antivenomic analysis of *D. typus* (Colubridae: Colubrinae) venom against CroFab™ antivenom (left) [134], antivenomic analysis of *P. papuanus* (Elapidae) venom against Australian antivenom (middle) [132], and antivenomic analysis of *B. diporus* (Viperidae: Crotalinae) venom against Butantan pentabothropic antivenom (right) [135]. Chromatograms labeled “a” display reference RP-HPLC separation of the venom proteins. Major protein classes identified in the different chromatographic fractions are highlighted (3FTx, three-finger toxin; PLA_2_, phospholipase A2; CRISP, cysteine-rich secretory protein; SVSP, snake venom serine proteinase; PIII-SVMP, snake venom metalloproteinase of class PIII; CTL, C-type lectin-like molecule; LAO, L-amino acid oxidase; VAP, vasoactive peptide; SVMPi, tripeptide inhibitor of SVMPs). Chromatograms “b” and “c” display, respectively, reverse-phase separations of the immunocaptured and the non-bound column fractions recovered from the immunoaffinity columns. Chromatograms “d” and “e” show, respectively, reverse-phase HPLC separations of the venom components recovered in the bound fractions of mock matrix and control IgG columns.

A number of experimental parameters that may potentially affect the performance of an antivenomics affinity column have been pointed out [133]. To minimize potential problems while maximizing the analytical capacity of antivenomics, the second generation protocol has been expanded. “Third generation antivenomics” is presented in this Special Issue. Figure 3 depicts the basic protocol for antivenomics and some examples of antivenomic analysis with antivenoms.

## 10. The Need of International Partnerships to Evaluate Antivenoms on a Global Basis and to Strengthen National Regulatory Agencies

As discussed in the previous sections, an integrated assessment of the preclinical efficacy of antivenoms should be designed based on the main pathophysiological effects induced by the venoms to be analyzed. Routine quality control sections in antivenom manufacturing laboratories and regulatory agencies are seldom prepared to perform the whole set of tests described above, and usually carry out only the neutralization of lethality test; in the case of regulatory agencies in many countries even this basic assay is not performed. This raises the possibility that some antivenoms might not be effective to neutralize the main toxic activities of venoms which they are supposed to neutralize, this becoming a critical issue from a public health perspective.

This situation can be confronted through international partnerships whereby a network of laboratories, mostly of research groups, set up the methodological platforms described in this review, and develop new methods for the assessment of antivenom efficacy. In this way, the antivenoms deployed in various regions of the world could be independently tested against relevant venoms of snakes inhabiting the regions where these antivenoms are distributed. This type of concerted effort will provide solid evidence of the preclinical efficacy, or inefficacy, of antivenoms. These sort of cooperative studies have been performed in Latin America over the years, and have provided valuable information on the scope of efficacy of antivenoms (see for example [36,40,69,136,137]. More recently, the efficacy of seven antivenoms distributed in sub-Saharan Africa has been studied against the venom of *Echis ocellatus* using neutralization tests and antivenomics [91,93]. Similar assessments have been performed in Asia [138], Australia [139] and Papua New Guinea [79,132]. It is necessary to expand these efforts in order to generate a complete picture of the preclinical spectrum of efficacy of antivenoms in the world. Although this is a formidable challenge, it is possible to achieve it through international partnerships under the coordination of the WHO or organizations such as the Global Snakebite Initiative [8,140].

The issue of developing expertise at national regulatory agencies on the preclinical efficacy of antivenom is of outmost relevance. There have been reports of acquisition, by national health authorities, of antivenoms which are not effective against local snake venoms due to the lack of appropriate analysis of the products (see for example [26]). In some cases, the only criteria for acceptance of an antivenom is the description of the product provided by the manufacturer, and its price. International efforts should be promoted to enhance the capacity of national regulatory agencies for the registration and acceptance of antivenoms. This has to include, at least, a basic understanding on antivenom manufacture and preclinical efficacy and, ideally, the establishment of national or regional quality control laboratories that assess the neutralization of venom lethality by antivenoms. The case of Brazil is noteworthy, whereby a national quality control laboratory, the Instituto Nacional de Controle de Qualidade em Saúde (INCQS), evaluates all antivenom batches produced by the various manufacturing laboratories on the country [35]. The improvement of the capacities of national regulatory agencies could be achieved through international workshops organized by the WHO and its regional offices, with the collaboration of international experts. The combination of strengthened national regulatory agencies and a thorough study of the preclinical efficacy of antivenoms by research networks will be significant leaps forward in the rigorous evaluation of antivenoms, as a way to ensure the safety and efficacy of these products for their clinical use.

## 11. Preclinical Testing Should Be Followed by Appropriate Clinical Assessment of Efficacy and Safety of Antivenoms

As discussed along this review, a meticulous preclinical testing of antivenom efficacy should be performed before the acceptance of a new antivenom, or the use of an existing antivenom in a new geographical setting. Nevertheless, demonstration of preclinical efficacy does not necessarily imply that an antivenom can be directly introduced for human use. As indicated in the WHO Guidelines of antivenoms [32,41], appropriate evaluation of antivenoms at the clinical level is necessary to ensure efficacy and safety. Even though the use of preclinical models described in this review are highly useful at predicting whether an antivenom is effective against a snake venom, there might be differences in the response of mice and humans to venoms and to antivenom treatment; hence, the performance of clinical trials is required as a final step in antivenom evaluation. 

Clinical trials of antivenoms are designed to address three main issues: (a) the optimal initial dose; (b) efficacy, i.e., the ability of antivenom to control the main clinical manifestations of envenoming; and (c) safety, i.e., the incidence and severity of early and late adverse reactions [32]. Dose-finding studies are usually followed by randomized, controlled trials in which the new antivenom is compared to another antivenom already in use or, in its absence, two doses of the new antivenom can be compared. Observational studies can be performed when assessing an existing antivenom for its use in a new geographical location. In addition, once an antivenom starts to be distributed, it is important to perform post-marketing surveillance, or phase IV studies, in order to detect aspects of efficacy and safety that had been unnoticed during regular phase II and III studies [32]. 

## 12. Concluding Remarks

Owing to the biochemical, toxicological and immunological complexity of snake venoms, and their highly diverse patterns of regional and ontogenetic variability, the evaluation of the preclinical efficacy of antivenoms represents a difficult but rather important task. A methodological platform exists for such analyses, based on a set of relatively simple laboratory techniques that combines in vivo and in vitro assays, including neutralization tests and immunochemical assessments, e.g., antivenomics. However, these assays are often implemented in research laboratories and not in the quality control laboratories of manufacturers and regulatory agencies. This raises the need for developing concerted international efforts to thoroughly analyze the spectrum of preclinical efficacy of many antivenoms currently in use in various regions of the world, as well as new antivenoms being developed.

Novel preclinical tests are required in order to evaluate venom activities that play a key role in the pathophysiology of some envenomings, such as assays for cardiovascular and renal toxicity. Likewise, the philosophy of the 3Rs needs to be actively promoted in the field of antivenoms to reduce the use of animals, systematically introduce the use of analgesics, and develop novel in vitro assays in substitution of in vivo tests. In parallel, national regulatory agencies need to be strengthened through workshops and training programs of diverse sorts, with the goal of ensuring that antivenoms being introduced and used in the clinics are safe and effective. These issues pose significant challenges to the research, regulatory, and public health international communities and organizations.
